# Understanding the Effect of a Changing Climate on the Re‐Emergence of Mosquito‐Borne Diseases in Vulnerable Small Island Nations: A Systematic Review

**DOI:** 10.1111/zph.13212

**Published:** 2025-02-05

**Authors:** Mohabeer Teeluck, Oyelola Adegboye, Stephan Karl, Diana P. Iyaloo, Emma McBryde

**Affiliations:** ^1^ College of Medicine and Dentistry James Cook University Townsville Queensland Australia; ^2^ Menzies School of Health Research Charles Darwin University Darwin Northern Territory Australia; ^3^ Public Health and Tropical Medicine, College of Public Health & Tropical Medicine James Cook University Townsville Queensland Australia; ^4^ Australian Institute of Tropical Health and Medicine James Cook University Townsville Queensland Australia; ^5^ Vector Biology and Control Division Ministry of Health and Wellness Curepipe Mauritius; ^6^ Centre for Clinical Research University of Queensland Brisbane Queensland Australia

**Keywords:** climate change, mosquito‐borne diseases, small islands, systematic review, vector‐borne

## Abstract

**Introduction:**

Drastic changes in meteorological variables due to climate change will likely have an implication on the proliferation of vectors such as mosquitoes. Extreme weather events may therefore promote the emergence/re‐emergence of mosquito‐borne diseases (MBDs) and potentiate the risk of endemicity, particularly, in small island nations.

**Method:**

A systematic review was chosen to methodically ascertain the knowledge gaps that exist in determining the influence of the changing climate on MBDs in small islands with vulnerable public health systems. This review was conducted using the PRISMA guidelines.

**Results:**

Following extraction of 600 articles from the databases, 16 studies were determined to meet the selection criteria. The majority of these research papers were from Sri Lanka (*n* = 9) while the remaining articles were distributed between islands in the Pacific and Atlantic Ocean. Several of these studies used regression modelling techniques to discuss the effect of multiple meteorological variables on the incidence of MBDs. A positive relationship was observed between temperature and the relative risk of MBDs in 72% of the papers. Rainfall enhanced dengue transmission in 84% of the studies included. All the articles discussing the effect of humidity illustrated a similar trend while wind speed was the only climatic variable demonstrating a negative relationship with MBDs.

**Discussion:**

Considering the intricate nature of the non‐linear exposure–response link is crucial when estimating the lagged effect of the changing climate on MBDs transmission. Other challenges associated with bias and confounders in the selected studies as well as meteorological data accessibility, were highlighted. Therefore, it was not possible to conclusively establish that the changing climatic variables do influence the spread of MBDs which accentuated the need for conducting further studies to illustrate the effect of changing weather variables on the incidence of MBDs, with an emphasis on vulnerable small island nations.

**Summary**

- Gathered evidence in this systematic review discussed the non‐linear exposure–response relationship between meteorological variables and mosquito‐borne diseases (MBDs) in small island nations, most vulnerable to public health challenges
- The gap in quality studies highlighted the urgent need to conduct further studies in small island nations to examine the association between weather variables and the incidence of MBDs.
- This review would encourage small island nations to strengthen their public health surveillance systems with the implementation of modelling techniques to forecast the effect of the changing climate on occurrence of MBDs.

## 1 Introduction

The World Health Organization (WHO) considers climate change the ‘single biggest health threat facing humanity’ (World Health Organization 2023). By the end of the 21st century, the global mean annual temperature is projected to increase by approximately 1.5°C above pre‐industrial levels, favouring extreme weather events with ramifications on rainfall patterns (Intergovernmental Panel on Climate Change 2019). It is expected that between 2030 and 2050, an additional 250,000 deaths will be attributed to climate sensitive diseases such as water‐borne and vector‐borne diseases (VBD) (World Health Organization 2023). The projected financial impact of climate change on global health outcomes will be between USD 2–4 billion per year by 2030 (World Health Organization 2023).

It is estimated that more than 17% of all infectious diseases are due to VBD, with over 700,000 fatalities recorded each year (World Health Organization 2020). The burden of VBD is significantly higher in tropical and sub‐tropical regions, with the tropics known to bear substantially greater economic‐related health inequalities (Callander and Topp 2020). Malaria is caused by the *Plasmodium* parasites, transmitted by anopheline mosquitoes, resulting in over 400,000 deaths being notified annually. Dengue is transmitted by *Aedes* mosquitoes and is the second highest burden VBD with more than 3.9 billion people from over 129 countries, being at risk of the disease (World Health Organization 2020; Cissé et al. 2022). Yellow fever, Zika, chikungunya and lymphatic filariasis are also known to cause significant morbidity and mortality, with health systems being disrupted disproportionately, particularly in low‐ and middle‐income countries (World Health Organization 2020).

The United Nations designated a group of Small Island Developing States (SIDS) as being the most affected by climate change. Small islands share social, economic and environmental profiles and are susceptible to similar public health challenges (United Nations 2023a). The vulnerability of SIDS to health impacts from climate change has also been highlighted by WHO at the COP23 conference (WHO UNFCCC 2017).

SIDS are susceptible to a broad range of climatic hazards due to drastic changes in mean temperature, precipitation and humidity. This will likely have implications for the propagation and behaviour of vectors such as mosquitoes and may lead to more frequent mosquito‐borne diseases (MBDs) outbreaks and the potential establishment of endemicity in SIDs (Thomas et al. 2020). Mavian et al. have reviewed the role of tropical islands as ‘hotspots’ for the global spread of MBDs to North American and European countries. Global trade and human movement from tropical island nations have contributed to the risk of the introduction of VBD to temperate countries (Mavian et al. 2018). Although these countries experienced mostly localised outbreaks, global warming will increase the occurrence of VBD and prolong their seasonality. This shift was modelled in a study by Ryan et al. (2019) which showed a significant increase in the transmission of *Aedes* MBDs using different climate projections.

Several reviews have been conducted to determine the association between climate change and climate‐sensitive diseases in small island nations in the Caribbean and the Pacific. In addition to conducting a scoping review for existing research relating to climate and health, Rise, Oura, and Drewry (2022) also identified eight studies discussing the effect of changing climate on VBD in the Caribbean. A scoping review was also carried out by Kim et al. (2022) to demonstrate quantitative and qualitative evidence of the health impacts of climate change in the Pacific Islands. Hosking et al. (2023) performed a systematic review to examine available literature in the Pacific Island Countries and Territories, linking the effect of climate to ‘water‐related infectious diseases’, which included Dengue and Malaria.

However, to date, no systematic review has evaluated the knowledge pertaining to the re‐emergence of MBDs for small islands, particularly those susceptible to the public health impact of climate change. To this end, this review aims to determine the association between meteorological variables and the frequency and transmission of MBDs outbreaks in small islands with vulnerable public health systems. Determining the exposure–response relationship will subsequently support policymakers from SIDS in making informed decisions regarding climate adaptation policy and vector control strategies.

## 2 Methodology

A systematic review was carried out using the PRISMA (Preferred Reporting Items for Systematic Reviews and Meta‐Analyses) guidelines (Page et al. 2021 and Table S1). The protocol was prepared and approved by the team members prior to conducting the systematic review. The protocol was registered on PROSPERO, Protocol ID (CRD42023455412).

### 2.1 Population

The UN categorised SIDS as island nations that are most at risk of the effects of changing climate (United Nations 2023a). However, for this review, this specific list was not appropriate because it contained developed island nations such as Singapore and a South American mainland country, Guyana (United Nations 2023b). Therefore, the present study classified the small island nations based on the Notre Dame Global Adaptation Initiative (ND‐GAIN) index, a comprehensive tool developed in collaboration with the Environmental Change Initiative (Notre Dame Global Adaptation Initiative 2023b). The ND‐GAIN index ranks countries based on their vulnerability and readiness as well as susceptibility and preparedness to the effects of climate change. For this review, the list was further stratified using the health score of each country for the year 2021. The health score describes the vulnerability of a country's public health system to climate change in terms of the spread of infectious diseases and provision of healthcare services (Notre Dame Global Adaptation Initiative 2023b).

Island nations that are most susceptible to the public health impact of climate change were selected based on their respective health score from the ND‐GAIN list of the top hundred countries (Tables S2 and S3). All island nations that are dependent territories of developed and/or high‐income countries were not included.

### 2.2 Search Strategy

The search strategy was described as shown in Table S2 and Table 1.

**TABLE 1:** Eligibility criteria and search strategy for the systematic review.

Inclusion criteria	Peer reviewed research articles associating MBDs propagation and/or outbreaks to climatic variables such as rainfall/precipitation, temperature, wind speed and humidity
	The targeted countries for this review consisted of only the most vulnerable island states according to their respective health scores from the top hundred list of countries from the ND‐GAIN index
	Only research articles from independent island nations were considered
	Observational studies (cross‐sectional studies, cohort studies and case–control studies), experimental studies (quasi‐experimental, Randomised controlled trials), spatiotemporal studies and time series analysis, including modelling studies and other published studies investigating the association between climate change and MBDs
	Human health studies
	The research studies included either one or more of the following MBDs, namely chikungunya, dengue, Zika, malaria, yellow fever and lymphatic filariasis
Exclusion criteria	Case studies, literature review, conference proceedings, viewpoint articles, review articles, project reports and theses
	Animal studies
	Research studies which did not consist of one of the targeted MBDs, named in the inclusion criteria
	Research articles from island nations which are dependent territories of developed and/or high‐income countries were omitted
Search strategy	Search period: 01 January 2000 and 31 October 2023
	Search limited to English articles only. Non‐English text was excluded
Study population	Bahamas, Barbados, Cabo Verde, Comoros, Dominica, Federated States of Micronesia, Grenada, Haiti, Jamaica, Kiribati, Maldives, Marshall Islands, Mauritius, Nauru, Papua New Guinea, Samoa, São Tomé and Príncipe, Solomon Islands, Sri Lanka, St Kitts and Nevis, St Lucia, St Vincent and the Grenadines, Timor‐Leste, Tonga, Tuvalu and Vanuatu

### 2.3 Eligibility Criteria

The following inclusion and exclusion criteria were used to identify research articles for this review and the inclusion search period used was between 01 January 2000 and 31 October 2023, as shown in Table 1.

### 2.4 Selection Process

Once the search was completed, the papers were then extracted from the databases and imported to Rayyan, a web and mobile app for systematic reviews (Ouzzani et al. 2016). Once uploaded onto Rayyan, the automated deduplication capability of the software was used to identify and remove duplicate articles, by comparing the title, author, journal and year (Ouzzani et al. 2016). Following this automated process, the remaining duplicates were manually removed by an investigator MT, and the titles and abstracts of the subsequent articles were reviewed to identify studies eligible according to the above selection criteria.

**FIGURE 1:**
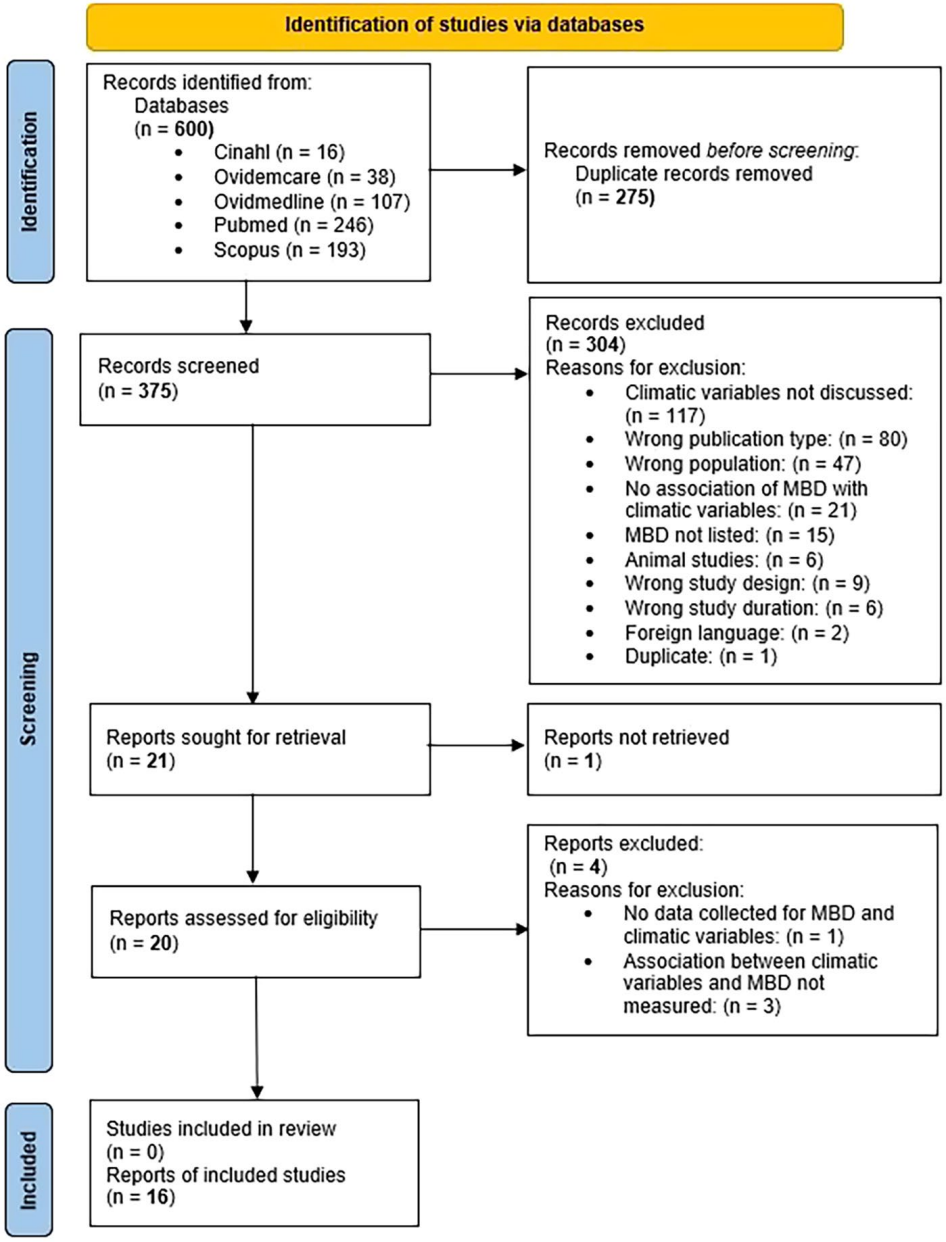
Flow chart illustrating the study selection process and results using the PRISMA 2020 guidelines.

Furthermore, the remaining articles were reviewed in full text, sorted by author's name in ascending order, and blinded using Rayyan by three of the main investigators (MT, EM and OA) who independently examined the 21 articles identified. Reasons for exclusion at both abstract and title screening as well as full‐text reviewing were included, as shown in the PRISMA chart (Figure 1).

### 2.5 Data Extraction

Data were extracted and collected on a spreadsheet (Table S4). The information collected includes bibliography details (author, title and publication year), study specifics (country, study period, study design, study projection and model type), meteorological factors (climatic variables included and time‐lag), other driving factors, disease characteristics (pathogen or disease, MBDs indicators), entomological characteristics (species and entomological indicators) and details of the dose–response relationship (exposure, outcome and direction/quantification of relationship). The data extraction process was first carried out by a single investigator (MT). Subsequently, EM and OA independently retrieved data from the studies. Any disagreements or discrepancies between the reviewers were discussed and resolved through consensus during a meeting.

### 2.6 Risk of Bias Assessment

Due to the limited availability of risk of bias (ROB) assessment tools for epidemiological modelling studies, it was necessary to adopt existing ROB techniques used in previously published systematic reviews. As a result, the ROB tool chosen for this study was originally adopted from Fone et al. (2003) and further modified by Harris et al. (2016), Aswi et al. (2018) and Odhiambo et al. (2020). The revised ROB tool consisted of eight‐point scoring criteria (Table S5), to assess the clarity of aims and objectives, study population, the methodology and appropriateness of the model and the results and conclusion of each study (Fone et al. 2003; Harris et al. 2016; Aswi et al. 2018; Odhiambo et al. 2020). The methodology for the screening questions consisted of a score ranging from 0 (poor) to 2 (good). The quality of each study was determined using the following designated categories; very high (> 13), high (11–13), medium (8–10) and low (< 8) (Aswi et al. 2018; Odhiambo et al. 2020). The scoring of the data was independently performed by four reviewers (MT, EM, OA and SK). Each reviewer assessed the data based on the predefined criteria (Table S6). Any disagreements or discrepancies in the scoring were discussed and resolved through consensus in a meeting.

## 3 Results

The database search identified 600 records, of which 275 were removed following the automated deduplication process and manual check for duplicates. The titles and abstracts of the remaining 375 articles were screened, after which 304 articles were excluded, and the reasons for exclusion are shown in Figure 1. Of the 21 full‐text articles assessed for eligibility using the above selection criteria, one article could not be reviewed as the full text was not available (Heidrich and Gotz 2022). Four records were excluded, of which three articles did not account for any measure of association between MBDs (Rodriguez‐Rodriguez et al. 2019; Surendran et al. 2018; Zhang et al. 2017) and meteorological factors, while one article did not contain data on climate and MBDs (Piyatilake and Perera 2020). Finally, 16 studies that met all selection criteria were included in this review (Table S4).

### 3.1 Descriptive Analysis

**FIGURE 2:**
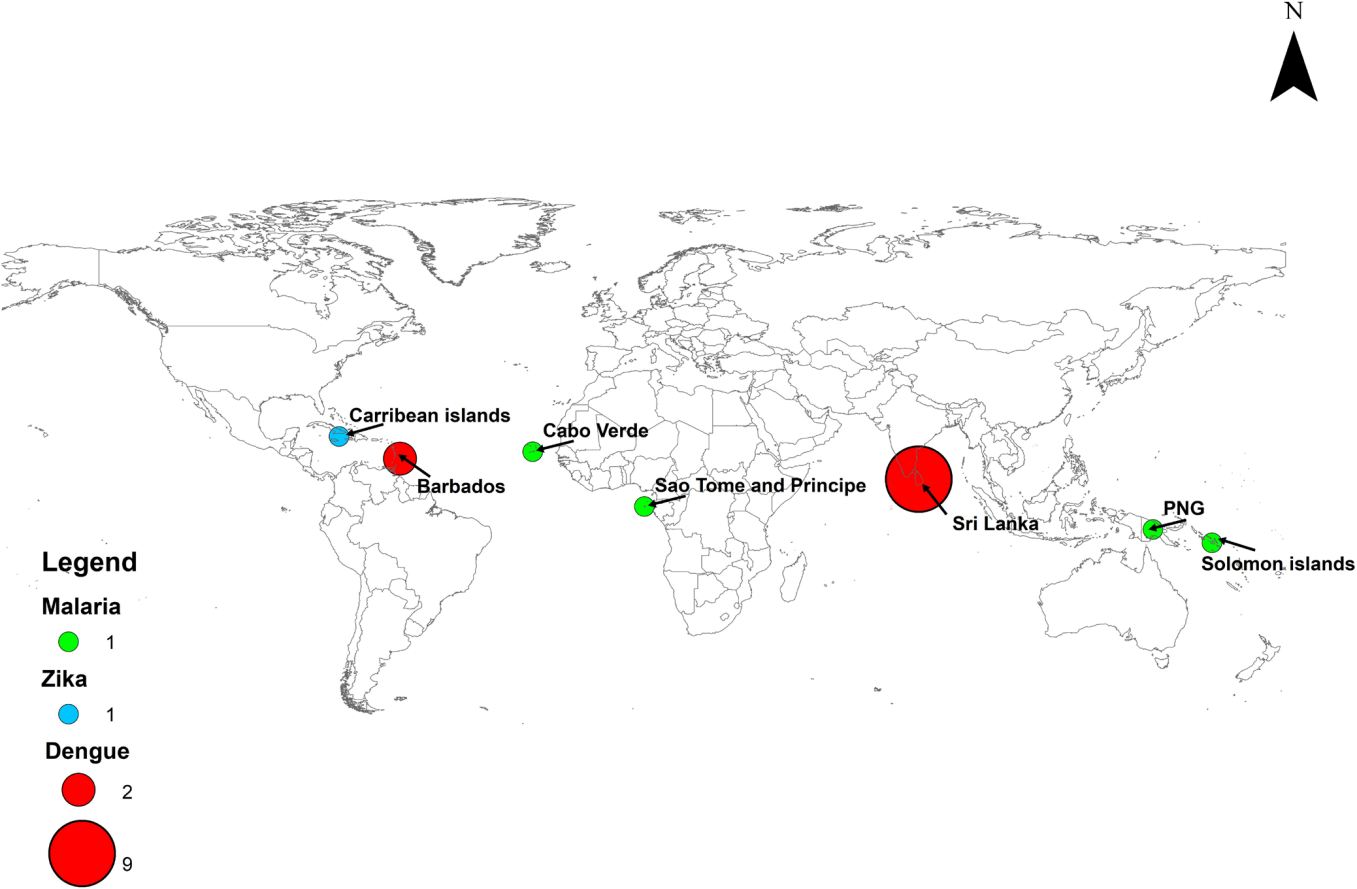
Distribution of articles categorised by region, countries and diseases.

Figure 2 shows the geographic distribution of the included research articles. Dengue was the disease under study in 11 out of 16 of the selected articles (Edussuriya, Deegalla, and Gawarammana 2021; Ehelepola et al. 2015; Ehelepola and Ariyaratne 2015, 2016; Sun, Xue, and Xie 2017; Talagala 2015; Faruk, Jannat, and Rahman 2022; Wijegunawardana et al. 2019; Wickramaarachchi, Perera, and Jayasinghe 2016; Amarakoon et al. 2008; Lowe et al. 2018). Of the 16 research papers (Sun, Xue, and Xie 2017; Talagala 2015; Edussuriya, Deegalla, and Gawarammana 2021; Ehelepola and Ariyaratne 2015, 2016; Ehelepola et al. 2015; Faruk, Jannat, and Rahman 2022; Wijegunawardana et al. 2019; Wickramaarachchi, Perera, and Jayasinghe 2016; Amarakoon et al. 2008; Lowe et al. 2018; Depina et al. 2019; Imai et al. 2016; Smith et al. 2017; Chen et al. 2019; Cunze et al. 2019) identified for the review, nine studies (over half) were from Sri Lanka in the Indian Ocean (Sun, Xue, and Xie 2017; Talagala 2015; Edussuriya, Deegalla, and Gawarammana 2021; Ehelepola and Ariyaratne 2015, 2016; Ehelepola et al. 2015; Faruk, Jannat, and Rahman 2022; Wijegunawardana et al. 2019; Wickramaarachchi, Perera, and Jayasinghe 2016). The impact of temperature and rainfall on dengue propagation was also investigated in two studies from Barbados in the Caribbean region (Amarakoon et al. 2008; Lowe et al. 2018). The association of weather characteristics with malaria was analysed in four out of 16 of the reviewed papers. These studies were carried out in Solomon Island and Papua New Guinea (PNG), in the Pacific as well as in Cabo Verde and São Tomé and Príncipe, in the Atlantic Ocean (Depina et al. 2019; Imai et al. 2016; Smith et al. 2017; Chen et al. 2019). The relationship between Zika virus transmission and climate was examined in a multi‐country study in the South and Central America region, including Haiti and Jamaica (Cunze et al. 2019).

Almost two thirds (10 out of 16) of the studies (Talagala 2015; Edussuriya, Deegalla, and Gawarammana 2021; Ehelepola and Ariyaratne 2015, 2016; Ehelepola et al. 2015; Wickramaarachchi, Perera, and Jayasinghe 2016; Amarakoon et al. 2008; Lowe et al. 2018; Imai et al. 2016; Smith et al. 2017) eligible for this review were retrospective time series analyses while four out of 16 used spatiotemporal analysis (Sun, Xue, and Xie 2017; Faruk, Jannat, and Rahman 2022; Depina et al. 2019; Cunze et al. 2019). A longitudinal study from São Tomé and Príncipe and a survey from Gampaha in Sri Lanka were also included in this review (Wijegunawardana et al. 2019; Chen et al. 2019). Four of the nine papers from Sri Lanka employed wavelet analysis for time series data to examine the association between meteorological variables and dengue (Ehelepola and Ariyaratne 2015, 2016; Ehelepola et al. 2015; Wickramaarachchi, Perera, and Jayasinghe 2016). Three of these four research papers were published by Ehelepola and Ariyaratne (2015, 2016) and Ehelepola et al. (2015).

Over half of the articles included in this review (*n* = 9) used regression techniques to model the variation observed between the dependent and response variables. This consisted of a study from Barbados, Solomon Island, PNG, Cabo Verde, São Tomé and Príncipe as well as four other studies in Sri Lanka (Sun, Xue, and Xie 2017; Talagala 2015; Edussuriya, Deegalla, and Gawarammana 2021; Faruk, Jannat, and Rahman 2022; Lowe et al. 2018; Depina et al. 2019; Imai et al. 2016; Smith et al. 2017; Chen et al. 2019). Six studies used various regression methodologies (Sun, Xue, and Xie 2017; Faruk, Jannat, and Rahman 2022; Depina et al. 2019; Imai et al. 2016; Smith et al. 2017; Chen et al. 2019), namely AutoRegressive Integrated Moving‐Average (*n* = 1) (Sun, Xue, and Xie 2017), negative binomial regression (*n* = 3) (Faruk, Jannat, and Rahman 2022; Imai et al. 2016; Chen et al. 2019) and multiple regression model (*n* = 1) (Depina et al. 2019). Other studies used the Distributed Lag Non‐Linear model (DLNM) to determine possible non‐linear and delayed associations between dengue incidence rates and weather variables (Talagala 2015; Lowe et al. 2018). Edussuriya, Deegalla, and Gawarammana (2021) chose the machine learning technique known as the Long Short‐Term Memory neural network to determine the association between climatic variables and dengue transmission.

Multi‐country studies were also selected for this review as they consisted of small island nations from the target population. Amarakoon et al. (2008) investigated the effect of temperature and precipitation on the Dengue epidemics in the Caribbean. Another study modelled the distribution of vectors and the risk of transmission of the Zika virus in South and Central America using a maximum entropy modelling approach. This paper was included in this review as data from Haiti and Jamaica on the habitat suitability of vectors as well as the effect of temperature, were analysed to determine its relationship with Zika propagation (Cunze et al. 2019).

### 3.2 Impact of Meteorological Variables on Dengue

#### 3.2.1 Temperature

Eleven articles discussed the effect of temperature on dengue transmission, of which nine were published in Sri Lanka, while the remaining two include Barbados as the study population, as shown in Table 2 and Figure S1 (Sun, Xue, and Xie 2017; Talagala 2015; Edussuriya, Deegalla, and Gawarammana 2021; Ehelepola and Ariyaratne 2015, 2016; Ehelepola et al. 2015; Faruk, Jannat, and Rahman 2022; Wijegunawardana et al. 2019; Wickramaarachchi, Perera, and Jayasinghe 2016).

**TABLE 2:** Summary of findings by geographical areas, study designs and main findings associated with dose–response relationship between MBDs and environmental factors.

Dengue publication	Country/geographical region	Study coverage/period	Study design	Model type	Exposure	Outcome	Change in outcome	Time lag	Dose–response relationship	Source of data (MBD/weather variables)
Temperature										
Sun, Xue, and Xie (2017)	Sri Lanka/Indian Ocean	2012–2016/monthly	Spatiotemporal cluster and hot spot analysis	ARIMAX (AutoRegressive Integrated Moving‐Average) model	Minimum, maximum and mean temperature	Dengue incidence	Increase in Colombo cluster and no change in other cluster	Unknown	Positive	MBD: Data from epidemiology unit, MoH. Weather: Department of meteorology
Talagala (2015)	Sri Lanka (Colombo district)/Indian Ocean	01/2009–09/2014	Time series	Quasi‐Poisson regression and distributed lag non‐linear model (DLNM)	Mean temperature (25°C–27°C)	Dengue relative risk	Increase	1–8 weeks	Positive	MBD: Epidemiological data from MoH. Weather: tutiembo.net/en (*Source*: Colombo local weather station)
					Mean temperature (> 28°C)	Dengue relative risk	Decrease	6–25 weeks	Negative	
					Maximum temperature	Dengue relative risk	Decrease	4–6 weeks	Negative	
Edussuriya, Deegalla, and Gawarammana (2021)	Sri Lanka/Indian Ocean	01/2010–03/2019/monthly	Time series	Long Short‐Term Memory neural network—LSTM	Temperature	Monthly dengue cases	Unknown		Strong association	MBD: Epidemiological data from MoH. Weather: Department of meteorology
Ehelepola et al. (2015) * (The interrelationship between dengue incidence and diurnal ranges of temperature and humidity in a Sri Lankan city and its potential applications)	Sri Lanka (Kandy)/Indian Ocean	2003–2012/weekly	Time series	Wavelet analysis	Weekly averages of diurnal ranges of temperature (Number of days with DTR > 10°C per week)	Dengue incidence	Decrease	3.3 weeks	Negative	MBD: Epidemiological data from MoH register. Weather: Two weather stations in Katugastota and Gannoruwa‐Peradeniya
					Weekly averages of diurnal ranges of temperature (Number of days with DTR < 10°C per week)	Dengue incidence	Increase	3 weeks	Positive	
					Weekly averages of diurnal ranges of temperature (Number of days with DTR < 8°C per week)	Dengue incidence	Increase	3.8 weeks	Positive	
Ehelepola and Ariyaratne (2016)	Sri Lanka (Colombo)/Indian Ocean	2005–2014/weekly	Time series	Wavelet analysis	Weekly averages of diurnal ranges of temperature (Number of days with DTR > 7.5°C per week)	Dengue incidence	Decrease	8 weeks	Negative	MBD: Weekly health ministry epidemiology reports. Weather: Department of Meteorology, Colombo
					Weekly averages of diurnal ranges of temperature (Number of days with DTR < 7.5°C per week)	Dengue incidence	Increase	8 weeks	Positive	
Ehelepola et al. (2015) * (A study of the correlation between dengue and weather in Kandy City, Sri Lanka (2003–2012) and lessons learned)	Sri Lanka (Kandy)/Indian Ocean	2003–2012/weekly	Time series	Wavelet analysis	Weekly minimum and maximum temperatures	Dengue incidence	Increase	5–7 weeks	Positive	MBD: Epidemiological data from MoH register. Weather: Two weather stations in Katugastota and Gannoruwa‐Peradeniya
Faruk, Jannat, and Rahman (2022)	Sri Lanka (26 regions)/Indian Ocean	2015–2019/monthly	Spatiotemporal analysis	Multivariate generalised linear negative binomial regression model	Temperature	Dengue incidence	No change		No association	MBD: Epidemiological data from MoH. Weather: Observed data from NASA Power Data Access Viewer
Wijegunawardana et al. (2019)	Sri Lanka (Gampaha District)/Indian Ocean	09/2014–09/2016/monthly	Survey	Pearson correlation analysis	Minimum and Maximum temperature	Monthly dengue cases	Decrease		Negative	MBD: Epidemiological data from Narangodapaluwa MOH area. Weather: Department of Meteorology, Colombo
Wickramaarachchi, Perera, and Jayasinghe (2016)	Sri Lanka (Urban Colombo)/Indian Ocean	2006–2012/weekly	Time series	Wavelet analysis	Maximum temperature	No. of weekly dengue cases	Unknown		Significant association	MBD: Data from epidemiological unit, Colombo Municipal Council. Weather: Department of Meteorology, Colombo
Amarakoon et al. (2008)	More focus on Trinidad & Tobago, Barbados and Jamaica (Only data from Barbados were considered as its study period 2000–2002)/Caribbean Ocean	2000–2002/monthly	Time series	Cross‐correlation analysis	Mean annual temperature	Dengue incidence	Increase	Few months	Positive	MBD: Epidemiological data from MoH Reports and Caribbean Epidemiology Centre in Trinidad. Weather: Meteorological stations
Lowe et al. (2018)	Barbados/Caribbean Ocean	06/1999–05/2016/monthly	Time series	Distributed lag non‐linear models	Minimum temperature (25.5°C)	Relative risk of dengue	Increase	2–3 months	Positive	MBD: Epidemiological data from surveillance unit, MoH. Weather: Two synoptic weather stations, Barbados
Rainfall										
Sun, Xue, and Xie (2017)	Sri Lanka/Indian Ocean	2012–2016/monthly	Spatiotemporal cluster and hot spot analysis		Mean Precipitation	Dengue incidence	Increase in Colombo cluster and no change in other cluster	Unknown	Positive	MBD: Data from epidemiology unit, MoH. Weather: Department of meteorology
Talagala (2015)	Sri Lanka (Colombo district)/Indian Ocean	01/2009–09/2014	Time series	Quasi‐Poisson regression and distributed lag non‐linear model (DLNM)	Precipitation (> 70 mm)	Dengue relative risk	Increase	1–5 weeks	Positive	MBD: Epidemiological data from MoH. Weather: tutiembo.net/en (*Source*: Colombo local weather station)
					Precipitation (20–50 mm)	Dengue relative risk	Increase	10–20 weeks	Positive	
					Precipitation (> 70 mm)	Dengue relative risk	Decrease	15–20 weeks	Negative	
Wijegunawardana et al. (2019)	Sri Lanka (Gampaha District)/Indian Ocean	09/2014–09/2016/monthly	Survey	Pearson Correlation analysis	Cumulative rainfall	Dengue cases	Increase		Positive	MBD: Epidemiological data from Narangodapaluwa MOH area. Weather: Department of Meteorology, Colombo
Amarakoon et al. (2008)	More focus on Trinidad & Tobago, Barbados and Jamaica (Only data from Barbados were considered as its study period 2000–2002)/Caribbean Ocean	2000–2002/monthly	Time series	Cross‐correlation analysis	Mean rainfall	No statistically significant association	Unknown		No association	MBD: Epidemiological data from MoH Reports and Caribbean Epidemiology Centre in Trinidad. Weather: Meteorological stations
Edussuriya, Deegalla, and Gawarammana (2021)	Sri Lanka/Indian Ocean	01/2010–03/2019/monthly	Time series	Long Short‐Term Memory neural network—LSTM	Average rainfall	Monthly dengue cases	Unknown		Strong association	MBD: Epidemiological data from MoH. Weather: Department of meteorology
Faruk, Jannat, and Rahman (2022)	Sri Lanka (26 regions)/Indian Ocean	2015–2019/monthly	Spatiotemporal analysis	Multivariate generalised linear negative binomial regression model	Precipitation	Dengue incidence	Decrease		Negative	MBD: Epidemiological data from MoH. Weather: Observed data from NASA Power Data Access Viewer
Lowe et al. (2018)	Barbados/Caribbean Ocean	06/1999–05/2016/monthly	Time series	Distributed lag non‐linear models	Positive Standard Precipitation Index (SPI) (> than average rainfall totals implying excess rainfall)	Relative risk of dengue	Increase	1–2 months	Positive	MBD: Epidemiological data from surveillance unit, MoH. Weather: Two synoptic weather stations, Barbados
					Negative SPI (< than average rainfall totals implying meteorological droughts)	Relative risk of dengue	Increase	5 months	Positive	
Ehelepola et al. (2015) * (A study of the correlation between dengue and weather in Kandy City, Sri Lanka (2003–2012) and lessons learned)	Sri Lanka (Kandy)/Indian Ocean	2003–2012/weekly	Time series	Wavelet analysis	Rainfall	Weekly dengue incidence	Increase	5–7 weeks	Positive	MBD: Epidemiological data from MoH register. Weather: Two weather stations in Katugastota and Gannoruwa‐Peradeniya
Wickramaarachchi, Perera, and Jayasinghe (2016)	Sri Lanka (Urban Colombo)/Indian Ocean	2006–2012/weekly	Time series	Wavelet analysis	Rainfall	No. of weekly dengue cases	Increase between 2010 and 2012		Positive	MBD: Data from epidemiological unit, Colombo Municipal Council. Weather: Department of Meteorology, Colombo
Relative humidity										
Sun, Xue, and Xie (2017)	Sri Lanka/Indian Ocean	2012–2016/monthly	Spatiotemporal cluster and hot spot analysis	ARIMAX (AutoRegressive Integrated Moving‐Average) model	Relative humidity	Dengue incidence	Increase in Colombo cluster and no change in other cluster		Positive	MBD: Data from epidemiology unit, MoH. Weather: Department of meteorology
Talagala (2015)	Sri Lanka (Colombo district)/Indian Ocean	01/2009–09/2014	Time series	Quasi‐Poisson regression and distributed lag non‐linear model (DLNM)	Relative humidity (65%–80%)	Dengue relative risk	Increase	10–18 weeks	Positive	MBD: Epidemiological data from MoH. Weather: tutiembo.net/en (Source: Colombo local weather station)
					Relative humidity (< 70%)	Dengue relative risk	Decrease	4–9 weeks	Negative	
Ehelepola et al. (2015) * (The interrelationship between dengue incidence and diurnal ranges of temperature and humidity in a Sri Lankan city and its potential applications)	Sri Lanka (Kandy)/Indian Ocean	2003–2012/weekly	Time series	Wavelet analysis	Weekly averages of diurnal ranges of humidity (Number of days with DHR > 20% per week)	Dengue incidence	Decrease	4 weeks	Negative	MBD: Epidemiological data from MoH register. Weather: Two weather stations in Katugastota and Gannoruwa‐Peradeniya
					Weekly averages of diurnal ranges of humidity (Number of days with DHR < 15% per week)	Dengue incidence	Increase	4 weeks	Positive	
Faruk, Jannat, and Rahman (2022)	Sri Lanka (26 regions)/Indian Ocean	2015–2019/monthly	Spatiotemporal analysis	Multivariate generalised linear negative binomial regression model	Humidity	Dengue incidence	Increase		Positive	MBD: Epidemiological data from MoH. Weather: Observed data from NASA Power Data Access Viewer
Ehelepola et al. (2015) * (A study of the correlation between dengue and weather in Kandy City, Sri Lanka (2003–2012) and lessons learned)	Sri Lanka (Kandy)/Indian Ocean	2003–2012/weekly	Time series	Wavelet analysis	Daytime and night‐time humidity	Dengue incidence	Increase	5–7 weeks	Positive	MBD: Epidemiological data from MoH register. Weather: Two weather stations in Katugastota and Gannoruwa‐Peradeniya
Wind speed										
Sun, Xue, and Xie (2017)	Sri Lanka/Indian Ocean	2012–2016/monthly	Spatiotemporal cluster and hot spot analysis	ARIMAX (AutoRegressive Integrated Moving‐Average) model	Wind speed	Dengue incidence	Decrease in Colombo cluster and no change in other cluster		Negative	MBD: Data from epidemiology unit, MoH. Weather: Department of meteorology
Talagala (2015)	Sri Lanka (Colombo district)/Indian Ocean	01/2009–09/2014	Time series	Quasi‐Poisson regression and distributed lag non‐linear model (DLNM)	Maximum sustained wind speed	Dengue relative risk	Decrease		Negative	MBD: Epidemiological data from MoH. Weather: tutiembo.net/en (Source: Colombo local weather station)
Edussuriya, Deegalla, and Gawarammana (2021)	Sri Lanka/Indian Ocean	01/2010–03/2019/monthly	Time series	Long Short‐Term Memory neural network—LSTM	Wind speed	Monthly dengue cases	Unknown		Strong association	MBD: Epidemiological data from MoH. Weather: Department of meteorology
Faruk, Jannat, and Rahman (2022)	Sri Lanka (26 regions)/Indian Ocean	2015–2019/monthly	Spatiotemporal analysis	Multivariate generalised linear negative binomial regression model	Wind speed	Dengue incidence	Increase but negative impacts in 2015 and 2016		Positive	MBD: Epidemiological data from MoH. Weather: Observed data from NASA Power Data Access Viewer
Ehelepola et al. (2015) * (A study of the correlation between dengue and weather in Kandy City, Sri Lanka (2003–2012) and lessons learned)	Sri Lanka (Kandy)/Indian Ocean	2003–2012/weekly	Time series	Wavelet analysis	Wind run (wind run = wind speed × duration)	Dengue incidence	Decrease	9 weeks	Negative	MBD: Epidemiological data from MoH register. Weather: Two weather stations in Katugastota and Gannoruwa‐Peradeniya
Mean visibility										
Talagala (2015)	Sri Lanka (Colombo district)/Indian Ocean	01/2009–09/2014	Time series	Quasi‐Poisson regression and distributed lag non‐linear model (DLNM)	Mean visibility > 14 km	Dengue relative risk	Increase		Positive	MBD: Epidemiological data from MoH. Weather: tutiembo.net/en (*Source*: Colombo local weather station)
					Mean visibility < 14 km	Dengue relative risk	Decrease		Negative	
Air pressure										
Faruk, Jannat, and Rahman (2022)	Sri Lanka (26 regions)/Indian Ocean	2015–2019/monthly	Spatiotemporal analysis	Multivariate generalised linear negative binomial regression model	Air pressure	Dengue incidence	Increase		Positive	MBD: Epidemiological data from MoH. Weather: Observed data from NASA Power Data Access Viewer
Sunshine hours										
Ehelepola et al. (2015) * (A study of the correlation between dengue and weather in Kandy City, Sri Lanka (2003–2012) and lessons learned)	Sri Lanka (Kandy)/Indian Ocean	2003–2012/weekly	Time series	Wavelet analysis	Sunshine hours	Dengue incidence	Increase	15 weeks	Positive	MBD: Epidemiological data from MoH register. Weather: Two weather stations in Katugastota and Gannoruwa‐Peradeniya
				Cross‐correlation analysis			Increase	6 weeks	Positive	
**Malaria publication**	**Country/Geographical region**	**Study coverage/period**	**Study design**	**Model type**	**Exposure**	**Outcome**	**Change in outcome**	**Time lag**	**Dose–response relationship**	**Source of data (MBD/Weather variables)**
Temperature										
Depina et al. (2019)	Cabo Verde/Atlantic ocean	01/2017–12/2017/monthly	Spatiotemporal	Multiple regression model	Mean temperature	Malaria incidence	Increase		Positive	MBD: Epidemiological data from NMCP and the National Surveillance Service, MoH. Weather: National Institute of Meteorology and Geophysics, Cabo Verde
Imai et al. (2016)	Papua New Guinea/Pacific Ocean	1996–2008/monthly	Time series	Generalised linear models (GLMs) with negative binomial distribution	Minimum temperature	Malaria cases	Decrease	0 months	Negative	MBD: Epidemiological data from the National Health Information System of PNG. Weather: PNG National Weather Service
					Minimum temperature	Malaria cases	Increase	2–3 months	Positive	
					Minimum temperature	Malaria cases	No effect	0–3 months	None	
					Minimum temperature	Malaria cases	Decrease	3 months	Negative	
Rainfall										
Smith et al. (2017)	Solomon Islands/Pacific ocean	1988–2013/monthly	Time series	Stepwise regression model	Heavy Rainfall	Malaria incidence	Decrease	0–2 months	Negative	MBD: National Vector‐Borne Disease Control Programme, MoH. Weather: Solomon Islands meteorological services
					Low rainfall	Malaria incidence	Increase	0–2 months	Positive	
Chen et al. (2019)	São Tomé and Príncipe islands/Atlantic ocean	2003–2016/monthly	Longitudinal study/survey	Negative binomial regression model	Rainfall up to 100 mm	Malaria incidence	Increase	1 month	Positive	MBD: Epidemiological data from MoH medical record system. Weather: Observed data from weather stations (Source: World Weather Online)
Depina et al. (2019)	Cabo Verde/Atlantic ocean	01/2017–12/2017/monthly	Spatiotemporal	Multiple regression model	Rainfall	Malaria incidence	Moderate increase		Positive	MBD: Epidemiological data from NMCP and the National Surveillance Service, MoH. Weather: National Institute of Meteorology and Geophysics, Cabo Verde
Imai et al. (2016)	Papua New Guinea/Pacific ocean	1996–2008/monthly	Time series	Generalised linear models (GLMs) with negative binomial distribution	Precipitation	Malaria cases	Increase in Madang but decrease in Eastern Highlands	1–2 months	Positive	MBD: Epidemiological data from the National Health Information System of PNG. Weather: PNG National Weather Service
Humidity										
Depina et al. (2019)	Cabo Verde/Atlantic ocean	01/2017–12/2017/monthly	Spatiotemporal	Multiple regression model	Relative humidity	Malaria incidence	Moderate increase		Positive	MBD: Epidemiological data from NMCP and the National Surveillance Service, MoH. Weather: National Institute of Meteorology and Geophysics, Cabo Verde
Wind speed										
Depina et al. (2019)	Cabo Verde/Atlantic ocean	01/2017–12/2017/monthly	Spatiotemporal	Multiple regression model	Wind speed	Malaria incidence	Decrease		Negative	MBD: Epidemiological data from NMCP and the National Surveillance Service, MoH. Weather: National Institute of Meteorology and Geophysics, Cabo Verde
**Zika publication**	**Country/geographical region**	**Study coverage/period**	**Study design**	**Model type**	**Exposure**	**Outcome**	**Change in outcome**	**Time lag**	**Dose–response relationship**	**Source of data (MBD/weather variables)**
Temperature										
Cunze et al. (2019)	27 countries, including Haiti and Jamaica	2015 and 2017	Maximum entropy approach	Correlative ecological niche modelling/maximum entropy modelling approach	Mean temperature of the warmest quarter	Zika transmission risk	Increase		Positive	MBD: Reported Zika cases sourced from WHO; CDC; GIDEON and PAHO. Weather: WorldClim used observed data to create bio10 (mean temperature of the warmest quarter)

Abbreviations: CDC, Centres for Disease Control and Prevention; GIDEON, Global Infectious Disease and Epidemiology Network; MBD, mosquito‐borne diseases; MoH, Ministry of Health; NASA, National Aeronautics and Space Administration; NMCP, National Malaria Control Programme; PAHO, Pan American Health Organization; PNG, Papua New Guinea; WHO, World Health Organization.

Five studies (three in Sri Lanka and two in Barbados) established a significant link between an increase in minimum and mean temperature and greater dengue transmission (Sun, Xue, and Xie 2017; Talagala 2015; Ehelepola et al. 2015; Amarakoon et al. 2008; Lowe et al. 2018), as shown in Table 2. A rise in mean temperature in Colombo, Sri Lanka, with a time lag of up to 8 weeks, caused an increase in dengue incidence (Talagala 2015). However, Talagala (2015) mentioned that a mean temperature of > 28°C had a delayed negative effect on dengue incidence by 6–25 weeks. Studies by Sun, Xue, and Xie (2017), Ehelepola et al. (2015) and Lowe et al. (2018) established that surges of dengue were associated with increase in minimum temperature which was observed at a mean lag between 7 and 10 weeks. Sun, Xue, and Xie (2017) identified that a rise in maximum temperature positively influenced the incidence of dengue while Ehelepola et al. (2015) illustrated this relationship coupled with a 5–7 weeks lag. On the other hand, Wijegunawardana et al. (2019) found a reduction in the monthly number of dengue cases with increasing maximum temperature in the Gampaha district, in Sri Lanka. Using DLNM, Talagala (2015) further demonstrated the relationship between maximum temperature and dengue incidence, considering a time lag between 4 and 6 weeks. Similarly, Lowe et al. (2018) investigated the association between minimum temperature and dengue transmission in Barbados. It was observed that the relative risk of dengue peaked at a minimum temperature of 25.5°C in comparison to when the minimum temperature was 21.5°C (Lowe et al. 2018). Furthermore, Amarakoon et al. used a lagged cross‐correlation analysis, which indicated a positive relationship between mean annual temperature and dengue incidence (Amarakoon et al. 2008).

Two studies explored the effect of diurnal ranges of temperature (DTR) on dengue incidence in the Kandy and Colombo, Sri Lanka (Ehelepola and Ariyaratne 2015, 2016). The wavelet analysis in Kandy demonstrated that the greater the number of days with DTR > 10°C per week, the lesser the transmission. In the case where there were more days with DTR < 10°C per week, dengue incidence was positively associated with the difference between minimum and maximum temperature (Ehelepola and Ariyaratne 2015). A similar observation was made for the experiment carried out in Colombo, with a DTR range of 7.5°C per week (Ehelepola and Ariyaratne 2016).

#### 3.2.2 Rainfall

Nine research (seven from Sri Lanka and two from Barbados) explained the impact of rainfall on the incidence of dengue as demonstrated in Figure S1 (Sun, Xue, and Xie 2017; Talagala 2015; Edussuriya, Deegalla, and Gawarammana 2021; Ehelepola et al. 2015; Faruk, Jannat, and Rahman 2022; Wijegunawardana et al. 2019; Wickramaarachchi, Perera, and Jayasinghe 2016; Amarakoon et al. 2008; Lowe et al. 2018). A positive correlation between precipitation and dengue was observed in six out of eight studies (Sun, Xue, and Xie 2017; Talagala 2015; Ehelepola et al. 2015; Wijegunawardana et al. 2019; Wickramaarachchi, Perera, and Jayasinghe 2016; Lowe et al. 2018) (Table 2). However, there are instances where too much rainfall led to a reduction in dengue incidence. This was discussed in a Sri Lankan study by Talagala, T., which described the impact of heavy rainfall, that is, greater than 70 mm, on dengue transmission. A positive trend in dengue relative risk was also recorded in this study after rainfall of 20–50 mm, and its associating time lag was 10–20 weeks. After a delayed period of 15–20 weeks following precipitation of > 70 mm, the relative risk of dengue was shown to drop significantly while a rise in dengue was revealed with a time lag of 1–5 weeks (Talagala 2015). Moreover, Faruk et al. established through an aggregate negative binomial regression model that rainfall significantly influenced dengue transmission, the risk of dengue incidence decreased by 3% for each unit (mm) of increase of precipitation (Odds:0.97, 95% CI: 0.97–0.99, *p* < 0.05) (Faruk, Jannat, and Rahman 2022). Furthermore, Lowe et al. (2018) explored the effects of floods and droughts on dengue transmission using the DLNM method. An increase in dengue transmission was observed in case of both flooding and drought events with their differing respective time lags of 1–2 and 5 months (Lowe et al. 2018).

#### 3.2.3 Relative Humidity

The effect of humidity on dengue transmission was investigated in five Sri Lankan studies as illustrated in Figure S1 (Sun, Xue, and Xie 2017; Talagala 2015; Ehelepola and Ariyaratne 2015; Ehelepola et al. 2015; Faruk, Jannat, and Rahman 2022). Four studies (Sun, Xue, and Xie 2017; Talagala 2015; Ehelepola et al. 2015; Faruk, Jannat, and Rahman 2022) showed a positive relationship between relative humidity and dengue incidence (Table 2). However, Talagala showed that a relative humidity of less than 70% (time lag: 4–9 weeks) was linked to a decrease in the relative risk of dengue. On the other hand, a relative humidity between 65% and 70%, with a time lag of 10–18 weeks, was associated with increased dengue transmission (Talagala 2015). Ehelepola et al. described the effect of diurnal ranges of humidity (DHR) on dengue incidence, which revealed a positive relationship with dengue with a greater number of days with DHR was < 15% per week. Conversely, if there were more days with DHR > 20% per week, disease transmission would be reduced (Ehelepola and Ariyaratne 2015). Another study by Ehelepola et al. (2015) in Kandy also highlighted the effect of humidity on dengue incidence using wavelet analysis. Increasing daytime and night‐time humidity favoured dengue transmission at a time lag of 5–7 weeks.

#### 3.2.4 Wind Speed

Five studies (Sun, Xue, and Xie 2017; Talagala 2015; Edussuriya, Deegalla, and Gawarammana 2021; Ehelepola et al. 2015; Faruk, Jannat, and Rahman 2022), published in Sri Lanka explored the influence of wind speed on dengue incidence (Figure S1). Three research papers established that increasing wind speed decreased dengue transmission (Sun, Xue, and Xie 2017; Talagala 2015; Ehelepola et al. 2015) (Table 2). However, the aggregate negative binomial regression model used by Faruk et al. demonstrated that dengue incidence increased by 6% for each unit of increase in wind speed. (Odds: 1.06, 95% CI: 1.02–1.11, *p* < 0.01). However, negative impacts were observed in the years 2015 and 2016 (Faruk, Jannat, and Rahman 2022).

### 3.3 Impact of Meteorological Variables on Malaria

#### 3.3.1 Temperature

Two studies, one from Cabo Verde and the other from PNG (Depina et al. 2019; Imai et al. 2016), recognised a link between temperature and malaria transmission (Table 2 and Figure S1). However, in the case of PNG, the research indicated that various time lags influenced the association between minimum temperature and the number of reported malaria cases (Imai et al. 2016). There was an increase in malaria transmission in the Western Highlands, Eastern Highlands and Madang provinces of PNG, with an increasing minimum temperature and a time lag of 2–3 months. Nonetheless, there was an immediate effect of minimum temperature (time lag of 0 month) leading to a decrease in the number of malaria cases in Western, Eastern Highlands and Madang provinces (Imai et al. 2016). The research paper from Cabo Verde demonstrated a positive association between mean temperature and the incidence of malaria (Depina et al. 2019).

#### 3.3.2 Rainfall

The four studies (Depina et al. 2019; Imai et al. 2016; Smith et al. 2017; Chen et al. 2019) that researched the effect of rainfall on the spread of malaria, showed a positive correlation between precipitation and the transmission of the disease (Table 2 and Figure S1). However, with a time lag of 0–2 months, low rainfall in Solomon Islands was shown to have a more significant association with malaria incidence in comparison with heavy rainfall (Smith et al. 2017). An increase in the incidence of Malaria followed precipitation up to 100 mm in São Tomé and Príncipe islands (Chen et al. 2019) while only a moderate rise in malaria transmission was seen in Cabo Verde after rainfall (Depina et al. 2019). On the other hand, while there was a significant reduction in malaria cases in Eastern Highlands due to rainfall, the opposite effect was observed in Madang, the other province in PNG (Imai et al. 2016).

#### 3.3.3 Relative Humidity and Wind Speed

The research paper from Cabo Verde associated a drop in malaria incidence with increasing wind speed, while a rise in relative humidity was shown to moderately increase disease transmission (Table 2 and Figure S1) (Depina et al. 2019).

### 3.4 Impact of Meteorological Variables on Zika

#### 3.4.1 Temperature

A multi‐country study by Cunze et al. (2019) used the correlative ecological niche modelling approach to examine the association between mean temperature and the spread of the Zika virus, as shown in Table 2 and Figure S1. It was revealed that an elevated mean temperature in the warmest quarter would increase the risk of transmission of Zika.

### 3.5 Impact of Other Meteorological Variables on MBDs

The full‐text screening of the articles that fit the inclusion criteria also revealed the ramifications of other meteorological variables on VBD transmission, such as mean visibility, air/atmospheric pressure and sunshine hours (Table 2) (Talagala 2015; Ehelepola et al. 2015; Faruk, Jannat, and Rahman 2022). Talagala (2015) established using the DLNM that mean visibility of > 14 km was associated with increased dengue transmission, while a negative relationship was observed when mean visibility was < 14 km (Talagala 2015). It was found following a search for other related studies on the web that there were limited research outputs available which explored the link between mean visibility and MBDs. The effect of atmospheric pressure on MBDs was also examined by Faruk, Jannat, and Rahman (2022) which conducted a spatiotemporal analysis using the multivariate generalised linear negative binomial regression model. Dengue incidence was shown to increase significantly by 46% for each unit (kPa) of atmospheric pressure (Odds: 1.46, 95% CI: 1.36–1.56, *p* < 0.01) (Faruk, Jannat, and Rahman 2022). The impact of sunshine hours on MBDs was explored in a Sri Lankan study by Ehelepola et al. (2015) and investigated using wavelet analysis, cross‐correlation coefficient and Spearman's rho. The wavelet methodology demonstrated a 15‐week delayed positive association between sunshine hours and MBDs. This relationship was confirmed by the cross‐correlation analysis in this study, which produced a coefficient of 0.125, thus, slightly favouring MBDs transmission at a time lag of 6 weeks (Ehelepola et al. 2015).

### 3.6 Summarising the Effect of Weather Variables on MBDs

**FIGURE 3:**
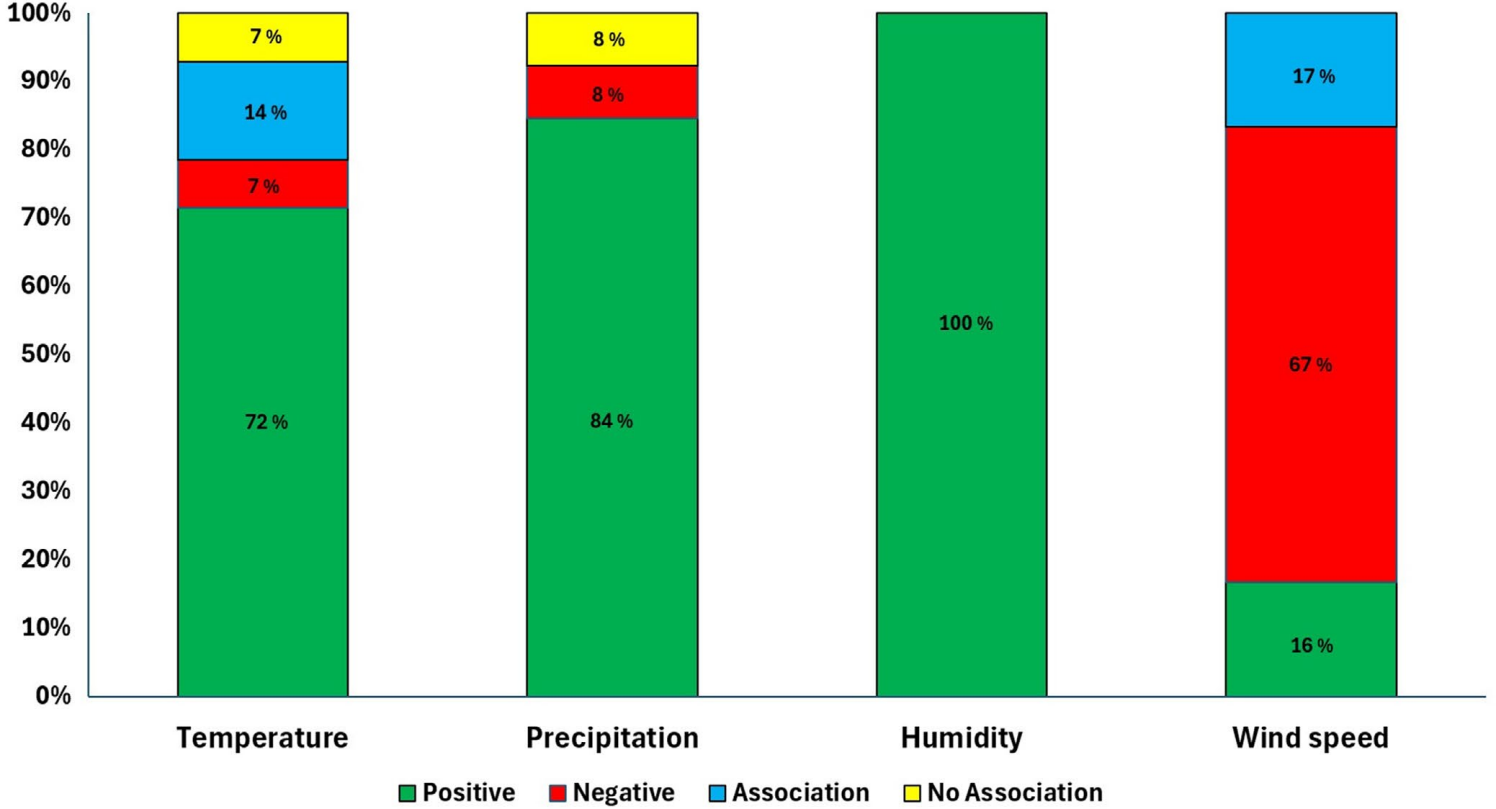
Hundred per cent stacked column chart illustrating the different types of association between weather variables and the transmission of MBDs.

In summation, the effect of more than one climatic variable on the transmission of VBD were described in 75% of the articles selected for this review (Sun, Xue, and Xie 2017; Talagala 2015; Edussuriya, Deegalla, and Gawarammana 2021; Ehelepola and Ariyaratne 2015; Ehelepola et al. 2015; Faruk, Jannat, and Rahman 2022; Wijegunawardana et al. 2019; Wickramaarachchi, Perera, and Jayasinghe 2016; Amarakoon et al. 2008; Lowe et al. 2018; Depina et al. 2019; Imai et al. 2016). 92.9% of the research papers demonstrated an association between temperature and MBDs (Sun, Xue, and Xie 2017; Talagala 2015; Edussuriya, Deegalla, and Gawarammana 2021; Ehelepola and Ariyaratne 2015, 2016; Ehelepola et al. 2015; Wijegunawardana et al. 2019; Wickramaarachchi, Perera, and Jayasinghe 2016; Amarakoon et al. 2008; Lowe et al. 2018; Depina et al. 2019; Imai et al. 2016; Cunze et al. 2019) while 7.1% showed no significant association (Faruk, Jannat, and Rahman 2022). Figure 3 revealed that approximately 72% of the research articles illustrated a positive effect between temperature and MBDs incidence (Sun, Xue, and Xie 2017; Talagala 2015; Ehelepola and Ariyaratne 2015, 2016; Ehelepola et al. 2015; Amarakoon et al. 2008; Lowe et al. 2018; Depina et al. 2019; Imai et al. 2016; Cunze et al. 2019). Notably, only one study by Faruk, Jannat, and Rahman (2022) denoted a negative association between MBDs and rainfall while approximately 84% of the studies demonstrated a positive relationship with precipitation (Figure 3). All six research papers demonstrated that increasing humidity favoured MBDs incidence, while a drop in humidity negatively correlated with VBD (Sun, Xue, and Xie 2017; Talagala 2015; Ehelepola and Ariyaratne 2015; Ehelepola et al. 2015; Faruk, Jannat, and Rahman 2022; Depina et al. 2019). Finally, among the six articles that demonstrated an association of wind speed and MBDs transmission, two thirds of the research outputs highlighted a negative relationship between wind speed and incidence of MBDs (Sun, Xue, and Xie 2017; Talagala 2015; Ehelepola et al. 2015; Depina et al. 2019).

### 3.7 Quality Assessment

Following the assessment of the reviewed papers, the total scores obtained varied between 8 and 16, as shown in Table 3. Over 30% of the studies (5 out of 16) were categorised as medium quality, while the remaining 11 papers, which represented almost 70% were either assigned a high (5 out of 16) or very high scores (6 out of 16). Generally, the quality of the studies was considered high, with a mean score and standard deviation of 12.3 and 2.72, respectively (Table 3). An overall mean score of 12.1 and a standard deviation (SD) of 2.4 confirmed the quality of the studies as high following further stratification of the scores in terms of meteorological variables (Table 4).

**TABLE 3:** Scores of publications using quality assessment tool.

ID	Author	Year	Study population	A&O	S&P	MS	MM	P, R&DS	QoD	PoR	IoR	Total scores	Grade
1	Smith et al.	2017	Solomon Islands	2	2	2	2	2	2	2	2	16	V. High
2	Sun et al.	2017	Sri Lanka	2	2	2	2	2	1	1	1	13	High
3	Talagala	2015	Sri Lanka	2	2	1	1	1	0	2	1	10	Medium
4	Wijegunawardana et al.	2019	Sri Lanka	2	2	1	1	0	1	1	2	10	Medium
5	Amarakoon et al.	2008	Barbados	2	2	1	1	1	1	1	2	11	High
6	Chen, Ying‐An et al.	2019	Sao Tome and Principe	1	2	1	1	1	0	1	1	8	Medium
7	Cunze et al.	2019	South and Central America	2	2	1	1	1	0	2	2	11	High
8	Depina et al.	2019	Cabo Verde	1	2	1	1	1	1	1	1	9	Medium
9	Edussuriya et al.	2021	Sri Lanka	2	2	2	2	2	2	1	2	15	V. High
10	Ehelepola; Ariyaratne	2015	Sri Lanka	2	2	2	2	1	2	1	2	14	V. High
11	Ehelepola; Ariyaratne	2016	Sri Lanka	2	2	2	2	1	2	1	2	14	V. High
12	Faruk et al.	2022	Sri Lanka	2	2	2	1	2	1	2	1	13	High
13	Ehelepola et al.	2015	Sri Lanka	2	2	2	2	2	0	1	2	13	High
14	Imai et al.	2016	Papua New Guinea	1	2	2	2	2	2	2	2	15	V. High
15	Lowe et al.	2018	Barbados	2	2	2	2	2	2	2	2	16	V. High
16	Wickramaarachchi, Perera and Jayasinghe	2016	Sri Lanka	2	1	2	2	0	0	0	1	8	Medium
			**Mean score**	1.8	1.9	1.6	1.6	1.3	1.1	1.3	1.6	12.3	High
			**SD**	0.40	0.25	0.50	0.51	0.70	0.85	0.60	0.50	2.72	

Abbreviations: A&O, aims and objectives; IoR, interpretation of results; MM, modelling methods; MS, model structure; P, R&DS, parameters, ranges and data source; PoR, presentation of results; QoD, quality of data; S&P, setting and population.

**TABLE 4:** Scores of publications using Quality Assessment tool, grouped by weather variables.

ID	Author	Temperature	Rainfall	Humidity	Wind	Grade	
1	Smith et al.		16				
2	Sun et al.	13	13	13	13		
3	Talagala	10	10	10	10		
4	Wijegunawardana et al.	10	10				
5	Amarakoon et al.	11	11				
6	Chen, Ying‐An et al.		8				
7	Cunze et al.	11					
8	Depina et al.	9	9	9	9		
9	Edussuriya et al.	15	15		15		
10	Ehelepola; Ariyaratne, K.	14		14			
11	Ehelepola; Ariyaratne, K.	14					
12	Faruk et al.	13	13	13	13		
13	Ehelepola et al.	13	13	13	13		
14	Imai et al.	15	15				
15	Lowe et al.	16	16				
16	Wickramaarachchi, Perera and Jayasinghe	8	8				
	**Mean score**	12.3	12.1	12.0	12.2	High	12.1
	**SD**	2.46	2.93	2.00	2.23		2.41

Abbreviations: A&O, Aims and objectives; IoR, Interpretation of results; MM, Modelling methods; MS, Model structure; P, R&DS, Parameters, ranges and data source; PoR, Presentation of results; QoD, Quality of data; S&P, Setting and population.

Significant gaps were also identified in several studies, especially in the case where over 30% of the reviewed articles did not address issues associated with data quality (Table 3). Moreover, considering the two research papers from the African island nations of Sao Tome and Principe and Cabo Verde, although the types of regression models were stated, the description of the methodology was less detailed (Depina et al. 2019; Chen et al. 2019). Additionally, the model description for four other studies were also deemed to be inappropriate by the reviewers, as shown in Table 3 (Talagala 2015; Wijegunawardana et al. 2019; Amarakoon et al. 2008; Cunze et al. 2019). Furthermore, results were not clearly presented in 50% of the papers (8 out of 16), as appropriate comparative analyses were missing, or the results were not aligned with the research questions. Furthermore, it is to be noted that 2 out of 16 studies did not report any parameters (Table 3).

## 4 Discussion

Small island nations have often experienced surges of VBD, and the changing tropical and/or sub‐tropical climate have contributed to MBDs recrudescence in these countries. Our review investigated the link between meteorological factors and the frequency and transmission of MBDs outbreaks in small islands with vulnerable public health systems. In this review, we identified only 16 articles that met the selection criteria, with over 55% of the studies being published in Sri Lanka. The remaining papers that were included were distributed between six islands nations in the Pacific, Caribbean and African islands. The use of modelling techniques is a key component in determining the effect of weather variables on MBDs associated with vector proliferation and control activities (Tjaden et al. 2018). Nonetheless, the lack of technical skills and limited availability of quality data have hindered the ability of SIDS countries to strengthen their modelling capabilities (Lowe et al. 2020). This study demonstrated the need to consolidate support to build analytical and modelling skills in small island nations in these regions, which also corroborated the findings from a study by Stewart‐Ibarra et al. (2019).

The majority of studies showed a positive correlation between MBDs and temperature. The results from this review were also consistent with the findings of other studies which explored the implication of temperature on the propagation of MBDs. Several models have demonstrated how rising temperatures could increase the global distribution of *Aedes* mosquitos, even in temperate countries (Ryan et al. 2019; Kamal et al. 2018). One study by Wijegunawardana et al. (2019) described a negative association of an increase in minimum and maximum temperature on dengue incidence. This study reported data quality concerns and may be an outlier for this reason. However, it also may be due to the non‐linear effect of temperature on mosquito growth and transmission efficiency.

The non‐linear correlation between temperature and MBDs is supported by in vitro, entomological experiments (Alto and Juliano 2001; Yang et al. 2009; Delatte et al. 2009). It was observed that there is an optimal temperature range for the reproduction and maturation of *Aedes* mosquitoes in the aquatic and adult phases, respectively (Alto and Juliano 2001; Yang et al. 2009; Delatte et al. 2009). Alto and Juliano (2001) observed that while controlled temperatures at 22°C and 26°C may promote the growth rate *Aedes albopictus* into adulthood, higher temperatures at 30°C will increase mortality rate. Yang et al. (2009) associated a temperature ranging between 15°C and 30°C to be ideal for *Aedes aegypti* mosquitoes to grow and proliferate, while a temperature above 35°C would inhibit their development. Additionally, Delatte et al. explored the association of temperature with the different stages of *Aedes albopictus* development in Reunion Island. It was found that *Aedes albopictus* optimum growth rate was recorded between 25°C and 30°C. However, negative growth was shown at a temperature of 35°C (Delatte et al. 2009). A model by Jia et al. (2019) also confirmed that mosquito development is significantly suppressed following a heat wave, defined as three consecutive days of temperature ≥ 35°C.

Most of the studies in this review showed a positive correlation between MBDs and rainfall. The influence of time lag between rainfall and a surge in MBDs is considered an important factor in determining the impact of rain on VBD incidence. This was explored by Talagala (2015), who established that following a period of 1–5 weeks, precipitation > 70 mm increased the relative risk of dengue. On the other hand, more than 70 mm of rainfall with a time lag of 15–20 weeks resulted in a drop in dengue transmission (Talagala 2015).

Several studies have also substantiated the results from this study in Sri Lanka, which described the non‐linear effect of rainfall on the proliferation of vectors and, consequently, the incidence of MBDs. This relationship varied significantly based on the magnitude and duration of precipitation (Hii et al. 2009; Chuang, Chaves, and Chen 2017; Lamy et al. 2023). A research paper in Singapore examined the effects of various lagged periods. Hii et al. used a Poisson regression model, which showed that a rise in relative risk of dengue was delayed by 5–12 weeks after rainfall of < 75 mm. However, dengue relative risk dropped with rainfall between 75 mm and 150 mm at a lagged stratum of 9–16 weeks, followed by a linear increase with precipitation above 150 mm (Hii et al. 2009). Moreover, Chuang, Chaves, and Chen (2017) adopted the DLNM method in southern Taiwan to exhibit the association between climatic variables (temperature and rainfall) and dengue transmission. It was ascertained that moderate to high rainfall had a positive delayed effect on the relative risk of dengue at 10 or 20 weeks. Low to moderate rainfall was also linked to a moderate increase in dengue transmission at a time lag between 0 and 5 weeks. In contrast, mosquito population distribution seems to be repressed with higher precipitation (68.2 mm), which peaked at time lags of 1 or 15 weeks (Chuang, Chaves, and Chen 2017). This ‘washout’ effect on mosquito proliferation due to heavy downpour in the January–February was also mentioned in a study in Reunion Island. Lamy et al. (2023) underscored the impact of intense rainfall flushed away the natural breeding sites of *Aedes albopictus* , affecting the development of mosquitoes from the aquatic phase to adulthood.

A significant association between relative humidity and MBDs was discerned in six articles (Sun, Xue, and Xie 2017; Talagala 2015; Ehelepola and Ariyaratne 2015; Ehelepola et al. 2015; Faruk, Jannat, and Rahman 2022; Depina et al. 2019). One study in Sri Lanka by Ehelepola et al. (2015) described the change in dengue incidence associated with the weekly average of diurnal range of humidity (Ehelepola and Ariyaratne 2015). Talagala (2015) also applied the DLNM method to explain the significance of the magnitude of relative humidity on dengue transmission in Sri Lanka (Talagala 2015).

The effect of relative humidity on the breeding, flight and feeding behaviours as well as the lifespan of *Aedes* mosquitoes, has been established (Brown et al. 2023; Juliano 2007). The exposure–outcome relationship between humidity and MBDs has also been appraised in several studies (Xiang et al. 2017; da Cruz Ferreira et al. 2017; Chen et al. 2010). For instance, research conducted in subtropical Guangzhou, China concluded, using the DLNM method, that a daily relative humidity of < 78.9% (95% CI: 73.3–84.5) was positively linked with the propagation of Dengue. However, Xiang et al. (2017) emphasised that dengue was negatively associated with extreme humid weather, that is, above 78.9%. The findings from the study in Guangzhou was also validated in another research output in subtropical, Porto Alegre in Brazil, which determined the effect of climatic factors on *Aedes aegypti* population. A Generalised Additive model (GAM) was fitted by da Cruz Ferreira et al. (2017) which also showed an adverse relationship between the mosquito population and relative humidity above 79%. Chen et al. elaborated a generalised estimating equations approach using a Poisson regression analysis to examine the association between *Aedes* mosquitoes and meteorological variables, including relative humidity, in Taiwan (Chen et al. 2010). The mean relative humidity in Taipei and Kaoshiung were estimated to be 74% and 72.5%, respectively. It was found that the relative humidity in both cities, positively influenced mosquito population density at a time lag of 3 months (Chen et al. 2010).

It was also noted that there was a substantial link between wind speed and MBDs in six papers (Sun, Xue, and Xie 2017; Talagala 2015; Edussuriya, Deegalla, and Gawarammana 2021; Ehelepola et al. 2015; Faruk, Jannat, and Rahman 2022; Depina et al. 2019) with the majority highlighting a negative relationship between wind speed and incidence of MBDs. Similar findings were observed in other studies investigating the effect of climatic factors on MBDs and their vectors in a tropical setting (Xiang et al. 2017; Gui et al. 2021). Gui et al. modelled the lag effect of wind speed on dengue transmission in Singapore using the ARIMA and DLNM model (Gui et al. 2021). The relative risk of dengue was observed to increase when the wind speed was between 5 and 7.52 km/h at a time lag of 1 week. On the other hand, a rise in wind speed to 9 km/h, following a delayed effect of 5 weeks, was negatively correlated with dengue transmission. Increasing wind speed to 13 km/h generally led to a drop in the relative risk of dengue at a time lag of 1 week (RR: 0.70, 95% CI: 0.57, 0.87) (Gui et al. 2021). Xiang et al. also discussed the inversely proportional relationship between wind speed and dengue incidence which was demonstrated at a wind velocity of > 10.7 m/s at a time lag of 0 (Xiang et al. 2017). The adverse effect of wind speed on dengue transmission was also observed through investigation of the *Aedes* mosquitoes trap rate in the tropical climate of Florida (Yang et al. 2021). Yang et al. (2021) determined that a mean average wind speed of 5.4 m/s would interfere with the *Aedes* mosquito population distribution and their host‐seeking behaviour, which would inherently affect dengue transmission.

The positive association of atmospheric pressure with MBDs found by Faruk, Jannat, and Rahman (2022) in this review, is also consistent with the conclusion from a study in Cuba (Fimia‐Duarte et al. 2019). Fimia‐Duarte et al. concluded that there is a strong positive correlation between atmospheric pressure and mosquito larval densities (Fimia‐Duarte et al. 2019). Additionally, a rise in dengue transmission associated with extended hours of sunshine was discussed by Elelepola et al. in a Sri Lankan study (Ehelepola et al. 2015). This was rationalised by the fact that longer days implied greater human activity outdoors especially at dusk and dawn, and therefore promoting vector–host contact rate (Ehelepola et al. 2015). On the other hand, overcast days might also intensify day mosquitoes' activities such as the *Aedes* spp. (Ehelepola et al. 2015; Gubler 1998). While it was expected that exposure to further sunshine hours would contribute to a rise in daytime temperature, studies from Thailand and South Taiwan have also shown the opposite effect (Lai 2018; Wongkoon, Jaroensutasinee, and Jaroensutasinee 2013). Lai (2018) and Wongkoon, Jaroensutasinee, and Jaroensutasinee (2013) mentioned that longer hours of sunshine was expected to raise the daily maximum temperature which would supress mosquitoes' population density due to a drop in mosquitoes' larval density.

We analysed 16 papers to assess the relationship between meteorological variables and MBDs in SIDS. We found that many studies show strong relationships. However, there were also many inconsistencies across studies. We also found non‐linear effects with a change in relationship at extremes of the variables especially with precipitation and temperature. Shocket et al. (2020) discussed this shift between positive and negative correlation with MBDs at either very low or high rainfall. Similarly, Mordecai et al. explained the unimodal nature of varying temperatures on the rate of transmission of MBDs (R_0_) using a ‘trait thermal based approach’ (Mordecai et al. 2019). Laboratory studies have shown that there is a complex interplay between meteorological variables and MBDs and therefore findings are likely to be highly specific to the local conditions in the study region.

A review by Mavian et al. (2018), also emphasised the environmental and socio‐economic burden shared by Indian Ocean, Pacific and Caribbean islands, making them particularly susceptible to the public health impact of MBDs. Islands are traditionally considered hot spots for VBD and extensive human movement due to tourism and trade has contributed towards the global emergence of MBDs outbreaks (Mavian et al. 2018; Cadavid Restrepo et al. 2021; Matthews et al. 2022). The COVID‐19 pandemic has particularly revealed the shortcomings of the public health systems of developing island nations. As a result, there is an expectation that SIDS countries will struggle in responding to disease outbreaks such as MBDs due to limited health infrastructures (Kousi et al. 2022).

Although these research articles underlined the associations between meteorological variables and the transmission of MBDs, it can be challenging to deal with bias and confounders in observational studies (Gilmartin‐Thomas, Liew, and Hopper 2018). Taking this into consideration, it is not possible to conclusively establish that the changing climatic variables do influence the spread of MBDs. Bias may be introduced in observational studies due to the limited availability of quality meteorological data especially in small island nations. Finally, each region has its own specificities, may have a notable impact on both mosquitoes and human behaviour, and thus, should be taken into consideration (Hawkes and Hopkins 2022).

### 4.1 Limitations

As explained in the methodology, the ND‐GAIN list of vulnerable island nations was used instead of the UN official SIDS list (Notre Dame Global Adaptation Initiative 2023b). This led to a study population that does not include all island states as defined by UN. Only independent island nations most susceptible to the impact of the changing climate on their public health systems were chosen from the ND‐GAIN list of top 100 most vulnerable countries (Notre Dame Global Adaptation Initiative 2023b). As a result, the impact of climate change on other island nations that are classified as dependencies or overseas territories is not considered. Therefore, studies from developed island states such as Singapore and Taiwan as well as dependent overseas territories such as Reunion Island, were explored further in the discussion section, and compared with the findings from SIDS to overcome this potential limitation (Hii et al. 2009; Chuang, Chaves, and Chen 2017; Lamy et al. 2023; Chen et al. 2010).

While there is the possibility that selection bias might have been introduced in this systematic review, we ensured a thorough selection process and risk of bias assessment, undertaken blind of other reviewer results, carried out independently by four reviewers. The selection process was blinded for the systematic review while the papers were also randomly allocated to the reviewers for quality assessment, which would support in addressing the risk of this bias. Moreover, exclusion of non‐English language articles in this review, may have also limit the generalisability of the findings while increasing the risk of bias of this study (Rockliffe 2022). Furthermore, consideration should be given to the risk of publication bias in this study due to omission of Grey literature which may result in missing out on key findings (Paez 2017).

For this systematic review, the meteorological factors that were considered in the search terms to evaluate the effect of the changing climate on MBDs were temperature, rainfall, humidity and winds. Moreover, the impact of other weather variables such as mean visibility (Talagala 2015), air pressure (Faruk, Jannat, and Rahman 2022) and sunshine hours (Ehelepola et al. 2015), from the selected research papers, were also measured during the data extraction process. Nonetheless, there were limitations associated with other complementary determinants of climate change and cross‐cutting issues such as changes in land use/urbanisation and population displacement/migration, that were not taken into consideration in this review (Speelman, Nicholls, and Safra de Campos 2021; Sobhee 2016). Still, the strength of this study was its specificity with regard to its study population by taking into account the vulnerability of the public health system and the socio‐economic aspects of the island nations when chosen for the inclusion criteria (Notre Dame Global Adaptation Initiative 2023a).

While a meta‐analysis was originally considered when developing the systematic review protocol, we determined that the studies selected in this review were not comparable. While the lack of homogeneity was due to different methodologies and outcomes being measured, other confounders need to be accounted for. For example, factors such as topology, culture (water storage practices) and other anthropological factors (Chumsri et al. 2019; Johnson and Sukhdeo 2013; Wilke et al. 2021) need to be considered in climate‐related disease studies. Additionally, the quality assessment of the selected articles for this review highlighted several limitations when assessing the appropriateness of the associated models. The poor description of the methodology and presentation of results in some of the reviewed studies also implied the potential presence of non‐reporting bias rendering a meta‐analysis inappropriate (Sharpe 1997).

## 5 Conclusion

The consequence of a changing climate on the emergence/re‐emergence of MBDs has been well established and is considered a global public health threat. This review was conducted to systematically examine available research articles and discuss the relationships between the shifting weather variables and MBDs outbreaks in small islands with susceptible public health systems.

The findings from this review highlighted significant associations between MBDs transmission and climatic factors, namely, temperature, rainfall, humidity and wind. However, further investigation using a meta‐analysis was not possible due to the lack of homogeneity of the studies. Moreover, the non‐linear exposure–response relationship should be considered when establishing the effect of changing meteorological variables on the distribution and spread of MBDs.

Furthermore, it was not possible to conclusively determine whether the changing climate effectively influenced MBDs transmission due to challenges associated with confounders and lack of access to quality weather data and published evidence, especially from SIDS (Lowe et al. 2020). Therefore, this systematic review highlighted the need to conduct further studies to examine the association between weather variables and the incidence of MBDs, with an emphasis on vulnerable small island nations.

Finally, in order to support informed policy decisions for vector control interventions, the development of climate‐related disease models for small island nations is key in forecasting the occurrence of MBDs. However, these models could be evaluated in further studies to take into account the unpredictability nature of confounding factors such as anthropogenic and socio‐economic factors as well resistance to insecticides and medication (Campbell‐Lendrum et al. 2015).

## Conflicts of Interest

The authors declare no conflicts of interest.

## Supporting information


**Table S1.** PRISMA 2020 Checklist.

**Table S2.** MeSH terms used in the search strategy for the systematic review.

**Table S3.** Ranking of small island nations according to their respective health scores.

**Table S4.** Data Extraction Sheet for Articles Selected in the systematic review.

**Table S5.** Risk of Bias Assessment tool for the systematic review.

**Table S6.** Scores for selected articles, assessed using the Risk of Bias Assessment Tool.

**Figure S1.** Geographical distribution of articles illustrating the proportion of studies associating mosquito‐borne diseases with the effects of meteorological variables.

## Data Availability

All data supporting the findings of this study are available within the paper and as Supporting Information. Table S3, the ND‐GAIN health score, from the Supporting Information have been obtained from this link: https://gain.nd.edu/our‐work/country‐index/download‐data/.
