# Supplementary material for: Understanding the Effect of a Changing Climate on the Re‐Emergence of Mosquito‐Borne Diseases in Vulnerable Small Island Nations: A Systematic Review
Source: Zoonoses Public Health. 2025 Feb 5;72(3):223–47. doi: 10.1111/zph.13212 (PMC11967312; doi:10.1111/zph.13212)
Supplement: Supplementary file 2 — Table S2. MeSH terms used in the search strategy for the systematic review. [file ZPH-72-223-s007.docx]

**Title:** Understanding the effect of a changing climate on the re-emergence of mosquito-borne diseases, in vulnerable small island nations: A systematic review

A query was carried out for indexed articles published in PubMed, Scopus, Emcare on Ovid, CINAHL Complete and Medline (Ovid) databases. The search strategy consisted of the following terms and was carried out by selecting “title, abstract and keywords” in the search bar of the above-mentioned databases, using the Boolean logic operator.

| **Item** | **Search terms** |
| --- | --- |
| 1. | "Mosquito borne disease*" OR "Vector borne disease*" OR Malaria OR Dengue OR Chikungunya OR "Lymphatic Filariasis" OR "Yellow fever" OR Zika OR "West Nile virus" OR "Rift Valley fever" OR "Ross River virus" |
| 2. | "Vector mosquito*" OR Mosquito* OR Aedes OR Anopheles OR Culex |
| 3. | Climate OR "Climat* change" OR "Climat* variab*" OR "Climat* factor" OR Meteo  OR Meteo* OR "Meteo* change" OR "Meteo* variab*" OR "Meteo* factor" OR Weather OR "Weather change" OR "Weather variab*" OR "Weather factor" OR Warm* OR Environment OR Temperature OR "Temperature change" OR Rain OR Rainfall OR Precipitation OR Humidity OR Wind OR "Wind speed" |
| 4. | "Bahamas" OR "Barbados" OR "Cabo Verde" OR "Cape Verde" OR "Comoros" OR "Dominica" OR "Federated States of Micronesia" OR "Grenada" OR "Haiti" OR "Jamaica" OR "Kiribati" OR "Maldives" OR "Marshall Islands" OR "Mauritius" OR "Nauru" OR "Papua New Guinea" OR "Samoa" OR "São Tomé and Príncipe" OR "Sao Tome and Principe" OR "Solomon Islands" OR "Sri Lanka" OR "St Kitts and Nevis" OR "St Lucia" OR "St Vincent and the Grenadines" OR "Timor-Leste" OR "Tonga" OR "Tuvalu" OR "Vanuatu" |
