# Supplementary material for: Understanding the Effect of a Changing Climate on the Re‐Emergence of Mosquito‐Borne Diseases in Vulnerable Small Island Nations: A Systematic Review
Source: Zoonoses Public Health. 2025 Feb 5;72(3):223–47. doi: 10.1111/zph.13212 (PMC11967312; doi:10.1111/zph.13212)
Supplement: Supplementary file 5 — Table S5. Risk of Bias Assessment tool for the systematic review. [file ZPH-72-223-s005.docx]

|  | **Criterion^[[1]](#footnote-1)^** | **Considerations^1^** | **Score considerations (0, none, 1, poor, 2, good)** |  |
| --- | --- | --- | --- | --- |
|  | **(A) Screening questions** |  |  | Definition,  Max 4 points |
| 1 | Does the paper clearly address aims and objectives? | Are the research questions and modelling objectives clearly defined? | 0 not stated  1 stated but vague  2 stated and focussed |  |
| 2 | Is the setting and population clearly defined? | Does the paper clearly state the setting (e.g. number of geographical location, number of MBD cases)? | 0 not stated  1 stated but vague  2 stated and focussed |  |
|  | **(B) Assessed the methodology and appropriateness of model** | |  |  |
| 3 | Is the model structure clearly described and appropriate for the research question? | Is there a description of model structure (prior for space, time or space-time)?  Does the model structure include covariates? | 0 not appropriate model structure, or no description of model  1 incomplete description  2 complete description | Model methods, Max 4 points |
| 4 | Are the modelling methods appropriate for the research question? | Were the modelling methods clearly described, and suited to the research question? | 0 not appropriate modelling method, or no description of method  1 incomplete description  2 complete description |  |
| 5 | Are the parameters, ranges and data source specified? | Are all parameters and their ranges reported? | 0 poorly reported  1 some information missing  2 complete reporting of parameters, ranges and data sources | Model inputs, Max 4 points |
|  |  | Are the data sources for parameters reported? |  |  |
| 6 | Is the quality of data considered? | Are data limitations discussed? | 0 no sources of uncertainty  1 partially addressed, and/or data inappropriate  2 fully addressed |  |
|  | **(C) Assessed the overall results and study conclusion** | |  |  |
| 7 | Have the results been clearly and completely presented? | Have the outcome values and their uncertainty ranges for each intervention/scenario been reported?  Do the results match the objectives?  Are sensitivity analyses clearly reported? | 0 Not reported, very unclear or not suited to research question  1 Stated, but ranges or planned sensitivity analyses missing and/or not directly aligned with research question  2 Values and ranges and planned sensitivity analyses reported and aligned with research question. | Results, Max 4 points |
| 8 | Are the results appropriately interpreted and discussed in context? | Are the results of the study discussed in context and generalisability considered? | 0 no discussion  1 some discussion but key points and/or limitations missed  2 full discussion of key points, limitations discussed |  |

Total score, Max 16

Very high > 13

High 11-13

Medium 8 -10

Low < 8

1. Adapted from Fone et al. (1) and Harris et al. (2). [↑](#footnote-ref-1)
